# Work–family conflict and anxiety among nurses of the maternal and child health institutions: the mediating role of job satisfaction

**DOI:** 10.3389/fpubh.2023.1108384

**Published:** 2023-06-30

**Authors:** Lipei Zhao, Jian Wu, Beizhu Ye, Clifford Silver Tarimo, Quanman Li, Mingze Ma, Yifei Feng, Xinghong Guo, Yalin Song, Minghan Zhang, Yuanyuan Fan

**Affiliations:** ^1^Department of Health Management, College of Public Health, Zhengzhou University, Zhengzhou, China; ^2^Henan Province Engineering Research Center of Health Economy & Health Technology Assessment, Zhengzhou, China; ^3^Department of Nursing and Health, School of Nursing and Health, Zhengzhou University, Zhengzhou, China

**Keywords:** nurses, anxiety, job satisfaction, work–family conflict, maternal and child health institution

## Abstract

**Introduction:**

Over the past decades, anxiety has garnered significant attention from nursing population. Investigations have centered on the correlation between work–family conflict (WFC) and anxiety as well as the link between job satisfaction and anxiety among nurses. However, the role of job satisfaction plays in the relationship between work–family conflict and anxiety remains relatively unexplored.

**Methods:**

In April 2021, a cross-sectional survey was conducted among nurses (*N* = 3,770) working at the maternal and child health institutions in Henan province, China. Multiple linear regression model was used to explore the factors associated with anxiety. Model 4 in Hayes’s PROCESS macro and Bootstrap method was performed to examine the mediating role of job satisfaction in the relationship between work–family conflict and anxiety.

**Results:**

The median (interquartile range) anxiety score was 5.00 (6.00). Work–family conflict was shown to be significantly correlated to job satisfaction (*r* = −0.517, *p <* 0.001) and anxiety (*r* = 0.457, *p* < 0.01). There was a strong negative correlation between job satisfaction and anxiety (*r* = −0.379, *p* < 0.01). The study also found that nurses aged 31–40 years, those with a junior college education (*p* = 0.001), those with an undergraduate or above education (*p* < 0.001), those who reported experiencing work–family conflict (*p* < 0.001), and those with lower job satisfaction (*p* < 0.001) were more likely to experience anxiety. Additionally, job satisfaction partially (a*b = 20.90%) mediated the relationship between work–family conflict and anxiety.

**Conclusion:**

The association between work–family conflict and anxiety among nurses in maternity and child health institutions was moderated by job satisfaction. Therefore, it is critical to enhance working conditions, minimize work–family conflict, and promoting job satisfaction among nurses may help to mitigate the negative effects of work–family conflict on anxiety.

## Introduction

Maternal and child health care institutions, have played an essential role in reducing maternal and child mortality and promoting the healthy development of women and children. Concomitant with the liberalization of the two-child and three-child policies in China, the substantial population size in China coupled with a severe shortage of nurses may result in heightened work–family conflict and reduced job satisfaction among them (1). Empirical evidence suggest that nurses who are employed in pediatric departments exhibits a greater propensity for mental health concerns in comparisons to their counterparts working in other departments (2). Stable mental conditions are of great importance for nurses to provide safe and quality services to their patients (3). Given the unique nature of duties carried out by nurses in maternal and child healthcare institutions, as well as the significance of their role in promoting the health and wellbeing of women and children, prioritizing work-related factors such as job satisfaction and work-family balance is crucial in fostering emotional and mental stability among nursing personnel, ultimately leading to the delivery of improved nursing care.

Anxiety can be described as a “state of uneasiness or apprehension resulting from the anticipation of a real or perceived threatening event or situation” (4). While low levels of anxiety can serve as a motivational force and elicit enthusiasm in an individual, prolonged exposure to such feelings may lead to adverse outcomes, such as impaired psychological wellbeing and diminished work performance (5). High anxiety levels have been shown to impair bodily functions, increase suicide ideation, and diminish quality of life (6). Multiple studies have revealed that the nursing community can be more anxious than the general population (5, 7, 8). Meanwhile, several factors in the work and family environment, such as role conflict and low job satisfaction, have been identified as risk factors for nurses’ poor mental and physical health (7, 9, 10). In order to enhance the efficacy and caliber of healthcare delivery for women and children, it is essential that measures be taken to lessen the anxiety of nurses working in maternity and child health institutions.

Work–family conflict can be defined as “a perception of insufficient energy or time to perform both the work and family roles successfully” (11). Due to the increased demand for human resources in medical disciplines, the burden of nurses has escalated, making them difficult to reconcile work and family duties (12). Previous studies in many countries confirmed that nurses faced great conflicts between work and family (12–14), which in turn has been reported to be associated with reduced levels of job satisfaction among them (3, 15, 16). Work–family conflict is anticipated to have an unfavorable impact on the mental health of individuals, which may consequently result in heightened level of anxiety (17–19). In addition, the effects of work–family conflict on mental health have been also confirmed in the context of occupational population in China (10, 17, 20).

Job satisfaction refers “a pleasurable emotional state arising from an individual’s awareness of his or her motivations at work” (21). People with high job satisfaction tend to exhibit a positive emotional state, whereas unsatisfied individuals are prone to show a negative emotional state and even have mental health symptoms with time going by, such as anxiety and depression (2). Therefore, job satisfaction has received much attention in the existing research since employees with higher job satisfaction tend to have higher job quality and efficiency at work (22), but also benefit from their personal physical and mental health (23, 24). Several studies conducted in China have shown that nurses’ job satisfaction is relatively lower than that in women working in other professions (1). Additionally, nurses exposed to overwork are more likely to be dissatisfied with multiple work factors and as a result, exhibit symptoms of mental health distress and anxiety (24–28). A study had proven that there was a significant dose–response relationship between dissatisfaction and anxiety, indicating that for each increase in the number of domains of dissatisfaction, the likelihood of symptoms increased (8).

Therefore, based on previous research findings, we proposed the following hypotheses.

*Hypothesis 1*: Work–family conflict had a direct path on anxiety.

*Hypothesis 2*: Job satisfaction mediated the relationship between work–family conflict and anxiety.

Despite the abundant researches on work–family conflict, job satisfaction, and anxiety, little is known about the factors associated with anxiety, the relationship between work–family conflict and anxiety and the potential mediators in this relationship among nurses in maternal and child health institutions. Therefore, the current study aimed to: (1) explore the independent factors associated with nurses’ anxiety; (2) examine the role of job satisfaction between work–family conflict and anxiety among nurses in maternal and child health institutions.

## Materials and methods

### Study design and participants

The cross-sectional study was conducted through “Wen Juan Xing” in April 2021, in Henan Province, China. Based on stratified cluster sampling, a total of 18 provincial cities in Henan Province were selected from one municipal to two counties (districts) levels of the maternal and child health institutions, and all health technicians in the selected institutions were invited to participate in the survey. All the studied participants were informed of the purposes of the studies before they filled in the questionnaires, then they filled the questionnaires after agreeing with the consent form. Inclusion criteria for this study included (a) adults aged 18 years or older, and (b) being able and willing to attend this study. In this study, a total of 3,770 nurses working in maternal and child health institutions were recruited for participation in a survey. Respondents with missing data for study variables were excluded, leaving a final sample of 3,183 nurses, representing an effective response rate of 84.43%. The vast majority of the nurses were female (99.25%), with those aged between 31 and 40 years constituting the largest group (42.63%). Moreover, most of the participants were married (81.81%), held an undergraduate or higher degree (51.62%), and were of junior rank (54.26%). Furthermore, a majority of the nurses (60.63%) were employed in secondary hospitals. The current study was approved by the Medical Ethics Committee of School of Public Health, Zhengzhou University (No: 2021-01-30-10). All study participants consented for participation in this study.

### Study measures

Participants were asked to report demographic information, including gender, age, marital status, education, professional title, and hospital grade.

The anxiety levels of the nurses were evaluated using the Generalized Anxiety Disorder 7-item (GAD-7) scale (29, 30), which consisted of questions such as “Over the past 2 weeks, how frequently have you experienced the following issues?” and “Feeling nervous, anxious or on edge?” Each of these questions was assessed using a four-point Likert scale, with response options ranging from “not at all” to “nearly every day. Each item was scored on a four-point Likert scale from 0 (not at all) to 3 (nearly every day). Possible scores for this scale range from 0 to 21. Previous studies in various Chinese populations have demonstrated good reliability and validity of the used scale (31, 32). The Cronbach’s alpha and Kaiser Meyer Olkin (KMO) coefficients of GAD-7 were 0.931 and 0.945, respectively.

Participants were asked to fill out the Chinese version of the Multidimensional Work–Family Conflict Scale to describe the subjective feelings of Work–Family Conflict (33). The scale comprised of 18 items that were categorized into two domains, namely work–family conflicts and family-work conflicts. Examples of items under the former category include “I cannot carry out some family activities due to the influence of work,” while the latter domain included statements like “Family life brings me anxiety and has an impact on my work performance.” Additionally, the scale incorporated three different forms of work–family conflict, which were time-based, strain-based, and behavior-based (34). Participants rated their level of agreement with each item on a five-point Likert scale 1 (very strongly disagree) to 5 (very strongly agree). The score ranges from 18 to 90 points. The Work–Family Conflict Scale had previous been extensively validated and applied across Chinese occupational populations (35, 36). In our study, the Cronbach’s alpha and KMO coefficients of the Work–Family Conflict Scale were both 0.933.

According to literature, seven questions were used to evaluate job satisfaction in our study (workload assignment, shift arrangement, work environment, promotion allocation, competitive mechanism, career development, and income; e.g., “How satisfied are you with the assignment of workload?”). Each question was assessed by using a five-point Likert scale: very satisfied, satisfied, neutral, dissatisfied, and very dissatisfied. Each item is scored from 1 (very dissatisfied) to 5 (very satisfied). The total score ranges from 5 to 35.

### Statistical analysis

**Figure 1:**
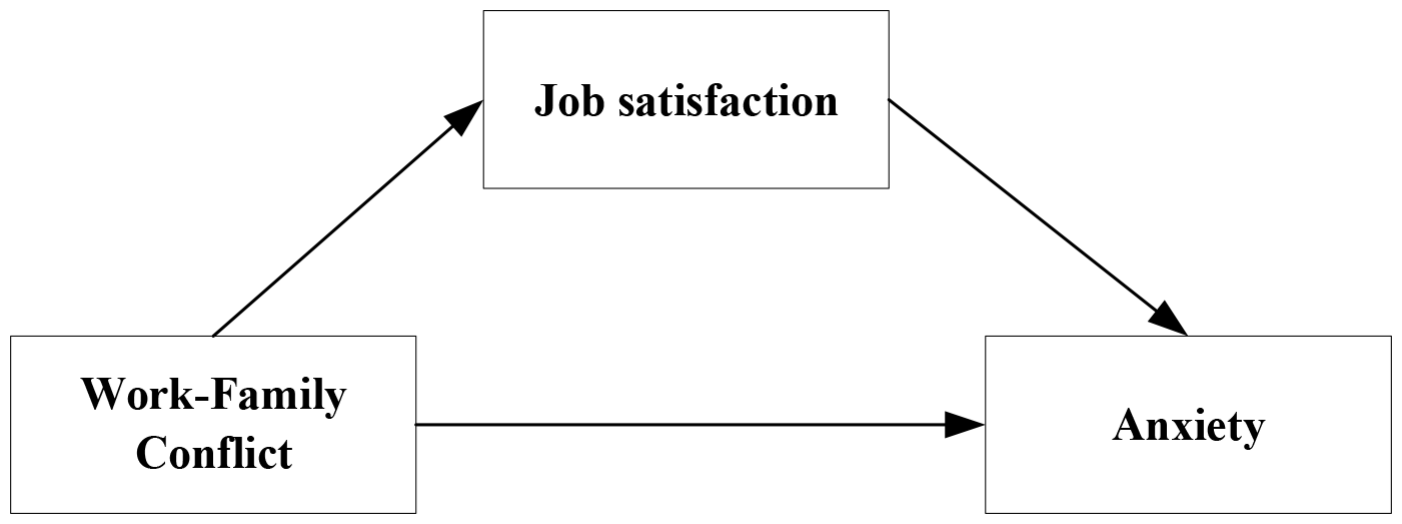
Hypothesized model.

The data were analyzed using the Statistical Package for Social Sciences (SPSS) software program, SPSS version 26.0 for the Windows platform. The normality of continuous variables was examined using Shapiro–Wilk test, which revealed that the variables under investigation, namely anxiety, work–family conflict, and job satisfaction, were non-normally distributed (*p* < 0.001). Therefore, median and interquartile range (IQR) were employed as descriptive statistics to represent these variables. Confirmatory factor analysis (CFA) was performed to test the reliability and validity of the job satisfaction scales. Earlier research suggested that a standardized factor loadings of at least 0.6, a Cronbach’s alpha of more than 0.7, a combined reliability (CR) exceeding 0.7, and an average variance extracted (AVE) greater than 0.5 signify sound convergent validity (37, 38). Categorical variables were expressed as frequency and percent. Mann–Whitney test and Kruskal-Wallis H test were conducted to compare the scores of WFC, job satisfaction, and anxiety. Differences between groups were considered statistically significant for *p* < 0.05. *Post hoc* multiple comparisons were carried out using the Bonferroni test, while Pearson’s correlation coefficient was employed to investigate the association among the study variables. Multiple linear regression analysis was used to determine the significant association between the anxiety and dependent variables (age, marital status, education level, professional title, hospital grade, WFC, and job satisfaction) with *p* < 0.05 in univariate analysis. In addition, the variance inflation factor was used to test for multicollinearity among the independent variables. The variance inflation factor was below the traditional cutoff value of 10, indicating that no collinearity was detected. Model 4 in Hayes’s PROCESS macro was used to test the hypothesized model (Figure 1) (39). First, sociodemographic variables (age, marital status, education level, professional title, and hospital grade) with *p* < 0.05 in univariate analysis were controlled in the subsequent analyses. Second, we defined work–family conflict as an independent variable (X) and anxiety as an outcome variable (Y); next, we tested whether the indirect path of X on Y was statistically significant; if 95% CI of the indirect path did not cross 0, there was a mediating path in the model; finally, we tested whether the direct path of X on Y was statistically significant. If 95% CI of direct path crossed 0, it implied that the mediator completely mediated the relationship between X and Y; if not, it indicated that the mediator partially mediated the relationship between X and Y.

## Results

### Confirmatory factor analysis

In this study, the job satisfaction scale demonstrated good reliability and validity with composite reliability and average variance extracted (AVE) of 0.942 and 0.699, respectively (Supplementary Table 1).

### Characteristics of participants

As shown in Table 1, the median (IQR) of anxiety symptoms, work–family conflict, and job satisfaction were 5.00(6.00), 48.00(15.00), and 27.00(8.00), respectively. Anxiety scores differed significantly according to age, marital status, education level, professional title, and hospital level. Additionally, work–family conflict and job satisfaction differed significantly according to age, marital status, education level, and professional title. Findings from the Bonferroni test and the detailed characteristics of work–family conflict, job satisfaction, and anxiety scores are shown in Table 1.

**Table 1:** Demographic characteristics of the study participants and the distribution of anxiety score.

Variables	*n* (%)	Work–family conflict median (IQR)	*p* value	Job satisfaction median (IQR)	*p* value	Anxiety median (IQR)	*p* value
All participants	3,183(100)	48.00(15.00)		27.00(8.00)		5.00(6.00)	
Gender			0.551^#^		0.143^#^		0.133^#^
Male	24(0.75)	50.50(14.00)		29.00(12.00)		5.00(6.00)	
Female	3,159(99.25)	48.00(15.00)		27.00(8.00)		2.50(7.00)	
Age (years)			0.001^※^		<0.001^※^		<0.001^※^
≤30	1,215(38.17)	48.00(16.00) ^a^		27.00(9.00) ^a^		5.00(7.00) ^a^	
31–40	1,357(42.63)	49.00(14.00) ^b^		27.00(9.00) ^b^		6.00(6.00) ^b^	
≥41	611(19.20)	46.00(14.00) ^a^		26.00(8.00) ^b^		5.00(7.00) ^b^	
Marital status			0.049^※^		<0.001^※^		0.023^※^
Unmarried	539(16.93)	48.00(17.00) ^a^		28.00(9.00) ^a^		4.00(7.00) ^a^	
Married	2,604(81.81)	48.00(14.00) ^a^		27.00(8.00) ^b^		5.00(6.00) ^a^	
Other	40(1.26)	50.50(9.00) ^a^		24.00(7.00) ^b^		7.00(8.00) ^a^	
Education level			<0.001^※^		<0.001^※^		<0.001^※^
Technical secondary school or below	182(5.72)	46.00(15.00) ^a^		27.00(7.00) ^a,b^		1.00(7.00) ^a^	
Junior college	1,358(42.66)	46.00(15.00) ^a^		27.00(9.00) ^b^		4.00(7.00) ^b^	
Undergraduate or above	1,643(51.62)	49.00(14.00) ^b^		26.00(8.00) ^a^		6.00(5.00) ^c^	
Professional title			0.001^※^		<0.001^※^		<0.001^※^
Junior title	1727(54.26)	48.00(16.00) ^a^		27.00(9.00) ^a^		5.00(7.00) ^b,c^	
Intermediate title	1,263(39.68)	48.00(13.00) ^b,c^		26.00(8.00) ^b,c^		6.00(6.00) ^a^	
Senior title	69(2.17)	48.00(10.00) ^a,c^		27.00(7.00) ^a,c^		6.00(5.50) ^a,b^	
Other	124(3.90)	45.50(16.00) ^a^		28.00(9.00) ^a^		3.50(7.00) ^c^	
Hospital grade			0.421^※^		0.113^※^		0.020^※^
Primary hospitals	647(20.33)	47.00(15.00)		27.00(8.00)		5.00(6.00) ^a^	
Secondary hospitals	1930(60.63)	48.00(14.00)		27.00(8.00)		5.00(7.00) ^a,b^	
Tertiary hospitals	606(19.04)	49.00(14.00)		27.00(8.00)		6.00(6.00) ^b^	

n, frequency; IQR, interquartile range. ^a–c^ The result of the Bonferroni test. a b, and c stand for different groups. There is no significant difference in vaccination rates within the same group.

# The category comparison was conducted by Mann–Whitney test.

※ The category comparison was conducted by Kruskal-Wallis H test.

### Correlation analysis

According to correlation analysis (Table 2), the results showed that the work–family conflict was significantly correlated to job satisfaction (*r* = −0.517, *p* < 0.01) and anxiety (*r* = 0.457, *p* < 0.01). Besides, job satisfaction was significantly and negatively correlated to anxiety (*r* = −0.379, *p* < 0.01).

**Table 2:** Pearson coefficients of correlations between the study variables in nurses.

Variables	Work–family conflict	Job satisfaction	Anxiety
Work–family conflict	1.000	-	-
Job satisfaction	−0.531^*^	1.000	-
Anxiety	0.442^*^	−0.361^*^	1.000

^*^p < 0.01.

### Multiple linear regression analysis of the factors of anxiety

Multiple linear regression analysis revealed that those aged 31–40 were more likely to experience anxiety compared to nurses aged 30 (*p* = 0.035). Those who had junior college (*p* = 0.001) and undergraduate or above (*p* < 0.001) were also more likely to experience anxiety. Additionally, based on multiple linear regression analyses, work–family conflict (*p* < 0.001), and job satisfaction (*p* < 0.001) were independent factors of nurses’ anxiety (Table 3).

**Table 3:** Results of multiple linear regression analysis with anxiety as the dependent variable.

Variables	B	SE	*β*	*t*	*p* value	95%CI
Age (years)						
≤30	ref.					
31–40	0.423	0.201	0.044	2.106	0.035	0.029 to 0.818
≥41	0.060	0.270	0.005	0.224	0.822	−0.468 to 0.589
Marital status						
Unmarried	ref.					
Married	−0.254	0.234	−0.020	−1.087	0.277	−0.712 to 0.204
Other	0.832	0.710	0.019	1.173	0.241	−0.559 to 2.223
Education level						
Technical secondary school or below	ref.					
Junior college	1.117	0.345	0.115	3.235	0.001	0.440 to 1.794
Undergraduate or above	1.954	0.352	0.204	5.544	<0.001	1.263 to 2.645
Professional title						
Junior title	ref.					
Intermediate title	−0.156	0.186	−0.016	−0.840	0.401	−0.520 to 0.208
Senior title	0.916	0.552	0.028	1.661	0.097	−0.165 to 1.998
Other	−0.066	0.395	−0.003	−0.168	0.867	−0.840 to 0.708
Hospital grade						
Primary hospitals	ref.					
Secondary hospitals	0.150	0.194	0.015	0.774	0.439	−0.230 to 0.530
Tertiary hospitals	0.430	0.245	0.035	1.757	0.079	−0.050 to 0.910
Work–family conflict	0.139	0.008	0.338	18.383	<0.001	0.125 to 0.154
Job satisfaction	−0.162	0.017	−0.175	−9.445	<0.001	−0.195 to − 0.128

### Testing for the mediator

**Figure 2:**
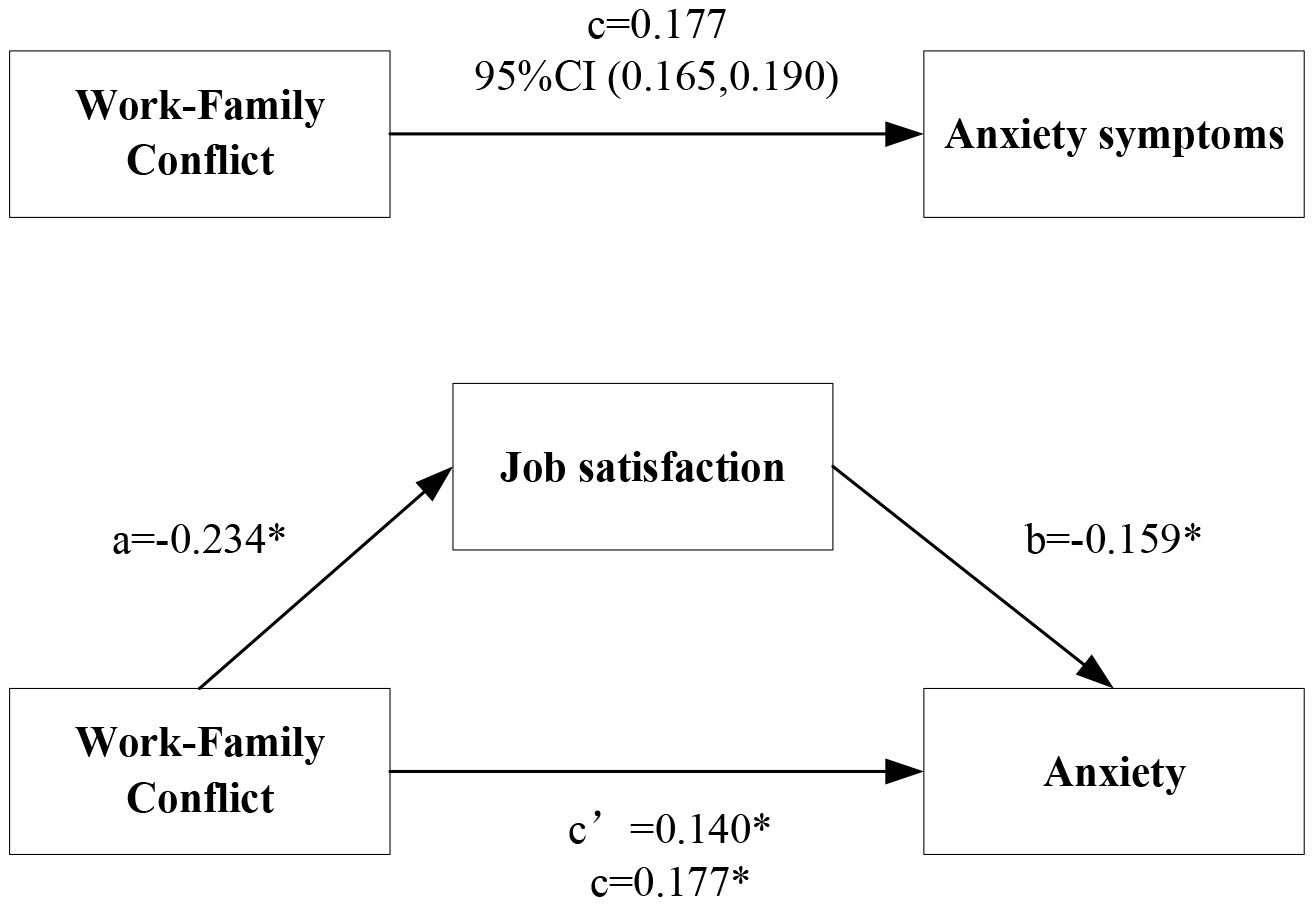
Path diagram of the relationships between work–family conflict, job satisfaction, and anxiety.

After controlling for age, marital status, education, professional title, and hospital grade as covariates, our analysis showed that the 95% confidence interval (CI) of the direct path between work–family conflict (0.126, 0.155) did not cross 0 with a CI of (0.028, 0.046), supporting the presence of a direct relationship between work–family conflict and anxiety. Therefore, hypothesis 1 was confirmed. Additionally, 95% CI (0.028, 0.046) in indirect path did not cross 0, indicating that job satisfaction partially mediated the relationship between work–family conflict and anxiety. Hence, hypothesis 2 was supported. We calculated the ratio of mediating path with total path was 20.90% (Table 4). Based on the result, we built a mediating model and path diagram of nurses’ work–family conflict and anxiety (Figure 2).

**Table 4:** Results of testing the mediating role of job satisfaction in the relationship between work–family conflict and anxiety.

	Effect	SE	*t*	*p* value	95%CI	Ratio of effect
Total path of X on Y(c)	0.177	0.007	27.187	<0.001	(0.165,0.190)	
Direct path of X on Y(c’)	0.140	0.008	18.519	<0.001	(0.126,0.155)	79.10%
Indirect path of X on Y(a^*^b)	0.037	0.005	-	-	(0.028,0.046)	20.90%

Controlling for age, marital status, education, professional title, hospital grade, X: work–family conflict, Y: anxiety, a: work–family conflict was negatively associated with job satisfaction; b: job satisfaction was negatively associated with anxiety after controlling for work–family conflict being negatively associated with job satisfaction; c: total path of work–family conflict on job satisfaction; c’: direct path of work–family conflict on anxiety after including job satisfaction as mediator; SE: standard error.

## Discussion

The current study explored the relationship between work–family conflict and anxiety and examined the mediation role of job satisfaction on this relationship among Chinese nurses working maternal and child health institutions. In our study, the median score of anxiety was 5.00 (IQR = 6.00), indicating a moderate level of anxiety (30). Additionally, job satisfaction partially mediated the relationship between work–family conflict and anxiety after controlling for sociodemographic variables (age, marital status, education level, professional title, and hospital grade) with *p* < 0.05 in univariate analysis.

We found that nurses aged 31–40 expressed a high level of anxiety than those under 30, in contrast to a study that found that nurses from 26 to 30 years of age had the highest prevalence of adverse mental health (2). Additionally, our results showed a significant difference in work–family conflict and job satisfaction among different age categories. One possible interpretation for the higher anxiety levels observed among nurses aged 31–40 is that, at this stage of life, they may have already established emotional stability and begun raising a family, leading to increased family obligations that may contribute to their anxiety, such as caring for children and older adults family members (17, 29). Meanwhile, occupational development is also at a critical period for them, making it challenging to balance family and work roles; and hence more likely to experience anxiety. Moreover, the regression analysis results showed that work–family conflict had a significant association with anxiety, which is consistent with previous studies (9, 14, 29, 40). Another finding from our study indicated a positive correlation between anxiety score and education level, aligning with prior research (25). It is probable that the reason for the increase in anxiety scores among nurses with higher education levels is due to the higher expectations they have for their professional growth. However, this may cause a gap between expectations and the reality of the high-pressure work environment, leading to anxiety. Additionally, nurses with different levels of education may have different opportunities for learning and career advancement, which could contribute to the observed variations in anxiety scores.

Additionally, our results suggest a mediating role of job satisfaction in the relationship between work–family conflict and anxiety. The possible reasons are discussed as follows. As per the previous literature, the profession of nursing is known to be highly demanding and stressful, leading to significant challenges related to work–family conflict among nurses (13, 14). Unfortunately, several studies have shown that work–family conflict was associated with lower job satisfaction among nurses (3, 15, 16). Resource conservation Theory (COR) holds that individuals have limited psychological, emotional, and material resources (41). Therefore, if nurses experience high levels of conflict or demands at work, they may use limited resources leaving fewer resources for family needs and vice versa. In balancing work and family roles, resources can be lost, mainly because the individual must use his/her resources to prevent further loss of resources (42, 43). Nurses exposed to these losses are likely to have lower job satisfaction (17, 18) and increased anxiety (10, 17, 18, 20, 41). Additionally, studies have shown that nurses who worked in the pediatric departments had a higher level of the mental problem compared with nurses in other departments (2).

On the other hand, the Spillover theory explains that, while there are temporal boundaries between work and family, there are no clear boundaries between emotions and behaviors, which can allow emotions and behaviors in one area to spill over into another (42, 44). According to the above two theories, nurses may develop negative emotions when they face conflicting roles between work and family. Suppose they are constantly exposed to role conflict, which will directly affect their mental health, harm work or family performance, and other aspects of life, thereby reducing job and family satisfaction. As such, dissatisfaction with jobs may translate to an increased risk for negative emotional states, anxiety, and depression. Additionally, studies confirmed a significant dose–response relationship between job dissatisfaction and anxiety (8). Above all, if nurses in maternal and child health institutions face great role conflict between work and family, it can not only directly increase anxiety level of nurses but also negatively affect job satisfaction. More importantly, lower job satisfaction significantly contributes to higher levels of anxiety (2).

Although this study provides the evidence necessary for nursing administrators to assist nurses in maternal and child health institutions, few limitations were identified. First, due to the cross-sectional design of the current study, we could not assess cause and effect relationships between work–family conflict and anxiety, studies to explore the causal relationship between these variables in cohort study will be necessary in the future. Second, the tool used in this study to evaluate job satisfaction is not a universal scale, so it may not be suitable for other countries and regions. Third, this study mainly examined the possible role of job satisfaction in the relationship between work–family conflict and anxiety, ignoring the mediating role of other situations on this relationship. Future studies should focus on their interactions and related boundary conditions, such as exploring the mediating role of self-efficacy, psychological capital, organizational support, and other variables on the mediation path. Future research can also further analyze the changing trend of nurses’ anxiety in different periods and the related mediating role.

## Conclusion

In conclusion, 31–40 years, junior college, undergraduate, or above, work–family conflict and job satisfaction were the related factors of nurses’ anxiety. Furthermore, job satisfaction partially mediated the relationship between work–family conflict and anxiety. It is therefore crucial to implement measures that can help reduce work–family conflict, increase job-satisfaction, and prevent or reduce anxiety symptoms among nurses.

## Data availability statement

The raw data supporting the conclusions of this article will be made available by the authors, without undue reservation.

## Author contributions

YuF, LZ, JW, and BY: conceptualization. JW, BY, LZ, QL, MM, YiF, XG, YS, and MZ: data curation. LZ: formal analysis and software. YuF, JW, BY, LZ, QL, MM, YiF, XG, YS, and MZ: investigation. YuF, LZ, and JW: methodology. JW: project administration. YuF, JW, BY: resources. LZ. YuF, JW, and BY: writing – original draft. YuF, CT, YS, and MZ: writing – review & editing.

## Funding

This study was funded by 2021 Postgraduate Education Reform and Quality Improvement Project of Henan Province (YJS2021KC07), Research on Monitoring, Evaluation and Assessment of Healthy Central Plain Action (20210232B), and Performance Evaluation of New Basic Public Health Service Projects in Henan Province (2020130B).

## Conflict of interest

The authors declare that the research was conducted in the absence of any commercial or financial relationships that could be construed as a potential conflict of interest.

## Publisher’s note

All claims expressed in this article are solely those of the authors and do not necessarily represent those of their affiliated organizations, or those of the publisher, the editors and the reviewers. Any product that may be evaluated in this article, or claim that may be made by its manufacturer, is not guaranteed or endorsed by the publisher.
