# Supplementary material for: Work–family conflict and anxiety among nurses of the maternal and child health institutions: the mediating role of job satisfaction
Source: Front Public Health. 2023 Jun 30;11:1108384. doi: 10.3389/fpubh.2023.1108384 (PMC10347398; doi:10.3389/fpubh.2023.1108384)
Supplement: Supplementary file 1 [file Table_1.DOCX]

| **Scale** | **Item** | **Standardized factor loading** | **Cronbach’s alpha** | **CR** | **AVE** |
| --- | --- | --- | --- | --- | --- |
| Job Satisfaction | workload assignment | 0.828 | 0.922 | 0.942 | 0.699 |
|  | shift arrangement | 0.833 |  |  |  |
|  | work environment | 0.847 |  |  |  |
|  | promotion allocation | 0.843 |  |  |  |
|  | competitive mechanism | 0.898 |  |  |  |
|  | career development | 0.897 |  |  |  |
|  | income | 0.689 |  |  |  |

**Table 1** **Confirmatory Factor Analysis**

Note. CR: composite reliability, AVE: average variance extracted.
